# The prevalence of musculoskeletal pain & its associated factors among female Saudi school teachers

**DOI:** 10.12669/pjms.306.5778

**Published:** 2014

**Authors:** Alsiddiky Abdulmonem, Algethami Hanan, Ahmed Elaf, Tokhtah Haneen, Aldouhan Jenan

**Affiliations:** 1 Alsiddiky Abdulmonem, Research Chair of Spinal Deformities, King Saud University, Riyadh, Saudi Arabia; 2 Algethami Hanan, Research Chair of Spinal Deformities, King Saud University, Riyadh, Saudi Arabia; 3 Ahmed Elaf, Research Chair of Spinal Deformities, King Saud University, Riyadh, Saudi Arabia; 4 Tokhtah Haneen, Research Chair of Spinal Deformities, King Saud University, Riyadh, Saudi Arabia; 5 Aldouhan Jenan, Research Chair of Spinal Deformities, King Saud University, Riyadh, Saudi Arabia

**Keywords:** Musculoskeletal, Pain, School teachers, Female, Saudi Arabia

## Abstract

***Objectives: ***To quantify the prevalence and identify the associated factors of musculoskeletal pain among Saudi female school teachers.

***Methods:*** An observational quantitative cross-sectional survey of female Saudi school teachers in five different areas of Saudi Arabia was carried out between August and October 2013. A self-administered questionnaire was used in which the items related to participants’ demographic information and pain information were included. A numeric pain rating scale was used for patient self-reporting of pain. Data analysis was carried out using SPSS Pc+ version 21.0 statistical software.

***Results:*** Four hundred and eighty six female school teachers responded to the survey. Severe Low back pain was reported by 38.1% of teacher, followed by knee pain (26.3%), heel (24.1%), shoulder (20.6%), upper back (17.7%), hip joint (16.5%),ankle (12.3%), neck (11.3%). Sever pain of elbow (5.6%) and wrist (7.4%) was the least reported. Pain affected work at school in 46.1% of school teachers. A combination of variables: body mass index, Vitamin D deficiency, teaching level, presence of chronic illness, were found to be significantly associated with musculoskeletal pain.

***Conclusion:*** The results of self-reported prevalence of musculoskeletal pain among female Saudi school teachers is useful to educate the school teachers for adequate care so as to prevent these pains. There is a need for the higher authorities to address this issue and implement intervention programs to alleviate the pain and suffering of these school teachers.

## INTRODUCTION

Musculoskeletal pain is a very common subjective complaint among working individuals. Evidence suggests that low back pain was due to minimum work place support and low job satisfaction.^1^ It is a global health issue resulting into chronic pain, functional impairment, frequent sick leaves and absences from work. Consequently, this equates with high economic-related implications burdened by less working hours, early retirement, less workforce and reduced productivity.^2^^,^^3^

Musculoskeletal pain ranks 6^th^ (shoulder pain), 7^th^ (neck pain) and 10^th^ (low back pain) as the most frequently reported health complaints among school teachers. Prevalence of musculoskeletal pain ranges from 40.4%^4^ to 69.3%.^5^ Among the reported areas of musculoskeletal pain among school teachers, the neck and shoulder,^6^^,^^7^ low back and the upper limbs are the most frequent painful sites because of their improper posture and high work load.^7^^,^^8^

Neck and shoulder pain occurs in 48.7% of school teachers correlated with prolonged standing, sitting and static posture.^9^ Low-back pain on the other hand is reported to occur in as much as 40% -75% of teachers.^10^ Upper limb pain is also frequent among teachers and was associated with heavier psychological demand with work movements in the upper limbs only.^11^

Several factors have been implicated with the high prevalence of musculoskeletal pain among school teachers. These included lifting of heavy load, prolonged sitting, improper posture, anxiety level, high job demand/workload, low peer/colleague support and poor mental status.^4^^,^^10^^,^^12^ Risk factors associated with significant musculoskeletal pain included gender, age, fixed posture, current working condition, and psychological framework of the patient.^7^^,^^13^ In other studies, lack of social confidence, poor social support, low level of education, poor work content, low job satisfaction, inadequacy of income, hard physical work, smoking, obesity, frequent lifting and improper posture, were the most important triggering factors.^8^^-^^10^

This study was conducted to determine the prevalence and risk factors of musculoskeletal pain among Saudi female school teachers.

## METHODS

An observational quantitative cross-sectional study was conducted among female Saudi school teachers. A self-administered questionnaire was personally distributed to 520 female school teachers in five different regions of the Kingdom. Questionnaires were distributed and collected over three months between August and October 2013. The questionnaire included participants’ demographic information (age. marital status, number of children, income level, educational level, position at work and number of pregnancy), low back pain information, work-related characteristics (including use of hand in work, number of work hours, number of teaching sessions), co-existing medical illnesses, lifestyle and habitual physical activity level (exercises and extracurricular activities, smoking), and effect of pain on daily activities including coping and self-administered treatment of pain.

A numeric pain rating scale^14^ was used for patient self-reporting of pain in some specified anatomical areas in the questionnaire. Rating of 0 will mean no pain, as mild pain, as moderate pain and as severe pain.

Content validity of the questionnaire was evaluated via two pilot tests among 20 female school workers. After these tests, we made minor revisions and finalized the initial version of the questionnaire. Analyses were performed on the responses of the 20 female respondents. The intraclass correlations coefficient of the respective domains in the questionnaire was 0.90 and 0.93, and the Cronbach’s alpha coefficients were 0.93 and 0.95, indicating adequate test-retest reliability and internal consistency for each domain of the questionnaire.

Data were encoded into a Microsoft Excel 2007 spreadsheet and was imported to SPSS Statistical Software version 21.0 (Chicago, Illinois, USA). Descriptive statistics (mean, standard deviation, and percentages) were used to describe the quantitative and categorical variables. One way analysis of variance was used to compare the mean value of quantitative variable in relation to categorical study variable. Pearson Chi-square was used to observe the association between the categorical study and outcome variables. A p value of <0.05 was considered as statistically significant.

## RESULTS

Out of 520 distributed questionnaires, 486 female school reaches responded and returned the filled-up survey forms with a response rate of 93.4%. Of these respondents, 255 (54.4%) in the age group of (30–39) years old. The Mean BMI of our study subjects was 27.6 ± 6.2 kg/cm^2^. There were 388 respondents (83.4%) who are married. Three hundred seventy four respondents had children. Majority of the respondents (74%) had a gross monthly family income of >9,000 Saudi riyals, 369 (92.3%) have a Bachelor’s degree. About 317 school teachers (66.7%) were having one housemaid at home. Four hundred thirty six respondents (93.6%) live within the city. (Table-I).

More than half of the respondents (n=266, 56.9%) were in service for more than 10 years. There were 139 (30.7%) elementary school teachers, 184 (40.6%) junior high school teachers and 107 (23.6%) senior high school teachers, the remaining 23 respondents (5.1%) were either teaching in either combinations of elementary, junior high and senior high school. The mean number of hours taught per day was 4.5 ± 2.9 and mean per week was 16.6 ± 6.0 hours. The vitamin D deficiency was reported by 200 (42.4%) teachers and 40.4% wakes up at night due to musculoskeletal pain. The school work was affected due to pain was reported by 199 (46.1%) and 277 (61.2%) were often had bad mood. About 334 (73.2%) mentioned that they often feel anxious. (Table-II) The self-reported musculoskeletal pain level showed prevalent of severe pain of low back among 185 (38.1%) school teachers, followed by 128(26.3%) teachers of knee pain and least was reported by 27 (5.6%) of elbow pain. (Table-III)

***Associated factors of musculoskeletal pain: ***The body mass index of school teachers is statistically significantly associated with the severity of heel pain, that is the mean body mass index of teachers suffering with severe heel pain is statistically significantly higher than the mean value of teachers with no pain and with mild & moderate heel pain. (F = 5.02; p=0.007). The severity of heel pain and hip joint pain are statistically significantly associated with marital status, where a higher proportion of teachers who were not single, were suffering with the severity of heel and hip joint pain. The four levels teaching (elementary, junior high school, senior high school and more than 2 levels) of these female school teachers is statistically significantly associated with the musculoskeletal pains of neck, knee, ankle and heel. The teachers, who were teaching junior high school level & senior high school level, were suffering with severe knee and ankle pain, which is statistically significant. (Table-IV).

**Table-I:** Distribution of Socio-demographic variables of female school teachers

**Variables**	**no.(%)**
**Age in years (n=469)**	
20-29 30-39 40 and above	71(15.1) 255(54.4) 143(30.5)
**Marital status (n=** **4** **66)**	
Single Married Divorced & Widow	50(10.7) 388(83.3) 28(6.0)
**Family Income per month in Saudi Riyals (n=457)**	
3000-5999 6000-8999 9000-11999 12000-14999 >= 15000	40(8.7) 78(17.1) 127(27.8) 100(21.9) 112(24.5)
**Educational Level(** **n=400)**	
Certificate/Diploma Bachelor’s degree Post graduate degree	18(4.5) 369(92.3) 13(3.2)
**Place of residence(n=466)**	
Within the city Outside the city	436(93.6) 30(6.4)
**No. of children (n=379)**	
1-3 children 4-6 children > 6 children	183(48.3) 170(44.8) 26(6.9)
**No. of pregnancies (n=380)**	
1-3 pregnancies 4-6 pregnancies > 6 pregnancies	156(41.1) 170(44.7) 54(14.2)
Housemaid at home (Yes/no) (n=471)	317(67.3)/154(32.7)

**Table-II:** Distribution of variables related to the work of female school teachers& their illness

**Variables**	**no.(%)**
**Level teaching at school(n=453)**	
Elementary Junior high school Senior high school More than 1 level	139(30.7) 184(40.6) 107(23.6) 23(5.1)
**Years at work (n=468)**	
1-5 6-10 11-15 16-20 >20	106(22.6) 96(20.5) 114(24.4) 88(18.8) 64(13.7)
**Way of going to school (n=464)**	
On foot (walking) By private car Private transport	12(2.6) 379(81.7) 73(15.7)
**No. of hours teach per week (n=421)**	
1- 10 hours 11-20 hours > 20 hours	65(15.4) 279(66.3) 77(18.3)
Furniture use at school is comfortable(Yes/No) (n=456)	163(35.7)/293(64.3)
Have extra-curricular activities (Yes/No) (n=443)	362(81.7)/81(18.3)
Presence of chronic illness (Yes/No) (n=473)	106(22.4)/367(77.6)
Presence of Vitamin D deficiency (Yes/No/Don’t know) (n=472)	200(42.4)/70(14.8)/202(42.8)
Wakes up at night because of pain(Yes/No)(n=438)	177(40.4)/261(59.6)
Pain affected work at school (Yes/No)( n= 432)	199(46.1)/233(53.9)
Often had a bad mood (Yes/No) (n=452)	277(61.2)/175(38.8)
Often feel anxious (Yes/No) (n=456)	334(73.2)/122(26.8)

**Table-III:** Distribution of musculoskeletal pain levels as reported by female school teachers

**Musculoskeletal parts**	**Levels of pain (n=486)**			
	**No pain**	**Mild**	**Moderate**	**Severe**
Neck Shoulder Elbow Wrist Upper back Low back Hip joint Knee Ankle Heel	210 (43.2) 198(40.8) 282(58.0) 289(59.5) 255(52.5) 161(33.1) 260(53.5) 179(36.8) 279(57.4) 214(44.0)	139(28.6) 94(19.3) 149(30.7) 122(25.1) 92(18.9) 70(14.4) 94(19.3) 109(22.4) 112(23.0) 100(20.6)	82(16.9) 94(19.3) 28(5.8) 39(8.0) 53(10.9) 70(14.4) 52(10.7) 70(14.4) 35(7.2) 55(11.3)	55(11.3) 100(20.6) 27(5.6) 36(7.4) 86(17.7) 185(38.1) 80(16.5) 128(26.3) 60(12.3) 117(24.1)

**Table-IV:** Association between self-reported Musculoskeletal pain and their BMI, Marital status & Level of teaching

**Type & level** **of pain**	**Body Mass Index**	**p-value**	**Marital Status**			**p-value**	**Level of Teaching**				**p-value**
			**Single**	**Married**	**Divorced & Widow**		**Elem.**	**Junior High**	**Senior High**	**More than 1 Level**	
**Neck** No pain Mild & Moderate Severe	---	----	----	----	----	---	57(41) 63(45.3) 19(13.7)	66(35.9) 100(54.3) 18(9.8)	49(45.8) 43(40.2) 15(14)	15(65.2) 8(34.8) 0(0)	0.039
**Hip joint** No pain Mild & Moderate Severe	**---**	-----	36(72) 11(22) 3(6)	198(50.4) 124(31.6) 71(18.1)	13(46.4) 10(35.7) 5(17.9)	0.048	---	---	---	---	----
**Knee** No pain Mild & Moderate Severe	----	----	----	----	----	----	41(29.5) 63(45.3) 35(25.2)	52(28.3) 72(39.1) 60(32.6)	45(42.1) 35(32.7) 27(25.2)	17(73.9) 1(4.3) 5(21.7)	<0.0001
**Ankle** No pain Mild & Moderate Severe	----	---	----	---	----	----	79(56.8) 40(28.8) 20(14.4)	90(48.9) 73(39.7) 21(11.4)	67(62.6) 26(24.3) 14(13.1)	17(73.9) 1(4.3) 5(21.7)	0.008
**Heel** No pain Mild & Moderate Severe	26.7(5.9) 27.4(5.8) 29.3(6.7)	0.007	30(60) 16(32) 4(8)	160(40.7) 129(32.8) 104(26.5)	11(39.3) 9(32.1) 8(28.6)	0.036	63(45.3) 45(32.4) 31(22.3)	65(35.3) 69(37.5) 50(27.2)	48(44.9) 30(28.0) 29(27.1)	15(65.2) 2(8.7) 6(26.1)	0.05

**Table-V:** Association between self-reported Musculoskeletal pain and the illness of female school teachers

*Type & level of pain*	*Vitamin D-Deficiency*		*p-value*	*Chronic illness*		*p-value*	*Feel anxious*		*p-value*	*Feel bad mood*		*p-value*
	*Yes*	*No*		*Yes*	*No*		*Yes*	*No*		*Yes*	*No*	
*Neck* No pain Mild & Moderate Severe	62(31) 106(53) 32(16)	36(51.4) 28(40) 6(8.6)	0.008	42(39.6) 44(41.5) 20(18.9)	155(42.2) 177(48.2) 35(9.5)	0.03	115(36.3) 159(50.2) 43(13.6)	64(52.5) 50(41) 8(6.6)	0.004	100(37.6) 126(47.4) 40(15)	78(44.6) 84(48) 13(7.4)	0.04
*Shoulder* No pain Mild & Moderate Severe	57(28.5) 86(43) 57(28.5)	34(48.6) 27(38.6) 9(12.9)	0.003	38(35.8) 35(33) 33(31.1)	148(40.3) 153(41.7) 66(18)	0.01	1056(33.4) 137(43.2) 74(23.3)	65(53.3) 38(31.1) 19(15.6)	0.001	92(34.6) 108(40.6) 66(24.8)	76(43.4) 72(41.1) 27(15.4)	0.037
*Elbow* No pain Mild & Moderate Severe	94(47) 89(44.5) 17(8.5)	47(67.1) 22(31.4) 1(1.4)	0.006	66(62.3) 28(26.4) 12(11.3)	203(55.3) 149(40.6) 15(4.1)	0.002	166(52.4) 129(40.7) 22(6.9)	82(67.2) 37(30.3) 3(2.5)	0.011	145(54.5) 101(38) 20(7.5)	103(58.9) 66(37.7) 6(3.4)	0.19
*Wrist* No pain Mild & Moderate Severe	101(50.5) 77(38.5) 22(11)	47(67.1) 22(31.4) 1(1.4)	0.012	70(66) 27(25.5) 9(8.5)	206(56.1) 134(36.5) 27(7.4)	0.11	166(52.4) 121(38.2) 30(9.5)	87(71.3) 30(24.6) 5(4.1)	0.001	145(54.5) 92(34.6) 29(10.9)	109(62.3) 59(33.7) 7(4)	0.03
*Upper back* No pain Mild & Moderate Severe	82(41) 69(34.5) 49(24.5)	42(60) 21(30) 7(10)	0.008	57(53.8) 26(24.5) 23(21.7)	186(50.7) 119(32.4) 62(16.9)	0.24	147(46.4) 102(32.2) 68(21.5)	77(63.1) 32(26.2) 13(10.7)	0.003	125(47) 82(30.8) 59(22.2)	98(56) 54(30.9) 23(13.1)	0.04
*Lower back* No pain Mild & Moderate Severe	48(24) 52(26) 100(50)	32(45.7) 23(32.9) 15(21.4)	<0.0001	33(31.1) 20(18.9) 53(50)	116(31.6) 120(32.7) 131(35.7)	0.008	87(27.4) 90(28.4) 140(44.2)	47(38.5) 43(35.2) 32(26.2)	0.002	77(28.9) 70(26.3) 119(44.7)	55(31.4) 62(35.4) 58(33.1)	0.03
*Hip joint* No pain Mild & Moderate Severe	89(44.5) 63(31.5) 48(24)	44(62.9) 21(30) 5(7.1)	0.004	57(53.8) 19(17.9) 30(28.3)	191(52) 127(34.6) 49(13.4)	<0.001	148(46.7) 103(32.5) 66(20.8)	81(66.4) 32(26.2) 9(7.4)	<0.0001	128(48.1) 79(29.7) 59(22.2)	101(57.7) 57(32.6) 17(9.7)	0.003
*Knee* No pain Mild & Moderate Severe	50(25) 74(37) 76(38)	36(51.4) 24(34.3) 10(14.3)	<0.0001	34(32.1) 29(27.4) 43(40.6)	133(36.2) 150(40.9) 84(22.9)	0.001	100(31.5) 117(36.9) 100(31.5)	52(42.6) 49(40.2) 21(17.2)	0.007	85(32) 94(35.3) 87(32.7)	68(38.9) 72(41.1) 35(20)	0.01
*Ankle* No pain Mild & Moderate Severe	98(49) 67(33.5) 35(17.5)	46(65.7) 21(30) 3(4.3)	<0.0001	61(57.5) 25(23.6) 20(18.9)	206(56.1) 122(33.2) 39(10.6)	0.03	159(50.2) 109(34.4) 49(15.5)	86(70.5) 30(24.6) 6(4.9)	<0.0001	143(53.8) 82(30.8) 41(15.4)	103(58.9) 57(32.6) 15(8.6)	0.11
*Heel* No pain Mild & Moderate Severe	69(34.5) 68(34) 63(31.5)	37(52.9) 27(38.6) 6(8.6)	<0.0001	41(38.7) 23(21.7) 42(39.6)	161(43.9) 132(36) 74(20.2)	<0.0001	115(36.3) 109(34.4) 93(29.3)	69(56.6) 36(29.5) 17(13.9)	<0.0001	104(39.1) 80(30.1) 82(30.8)	79(45.1) 66(37.7) 30(17.1)	0.005

The vitamin D-deficiency among female school teachers is highly statistically significantly associated with all the parts of musculoskeletal pain (neck, shoulder, elbow, wrist, upper back, lower back, hip joint, knee, ankle and heel). A higher proportion of teachers who had vitamin D-deficiency were having severe musculoskeletal pain when compared with the proportion of teachers who did not have vitamin D-deficiency which is statistically significant. The self-reported information of presence of chronic illness among them is also statistically significant with the severity of musculoskeletal pain of all parts except the parts of wrist and upper back. The presence of feeling often anxious and feeling often bad mood among school teachers was also statistically significantly associated with all part of musculoskeletal pain expect elbow and ankle parts not associated with the presence of bad mood.(Table-V).

## DISCUSSION

The rise of overused and tired body including the bones, joints and muscles results in musculoskeletal pain. Most of the time, it is work-related and can be acute or chronic. It has been shown in studies that a chronic persistent musculoskeletal pain consequently results into absences from employees, ill health retirement and even economically burdening employers and institutions.^2^^,^^3^ School teachers, especially elementary and pre-college school teachers, are very vulnerable to musculoskeletal pain because of their nature of work. They spend most of the time standing and moving around to monitor progress in teaching and ensuring their students comprehensibility on the subject matter. The process is repetitive, unlike in most college institutions where teaching is more modular than a demonstration-type of traditional teaching.

Our study showed a high prevalence of musculoskeletal pain particularly low back pain. Our findings showed that the prevalence of low back pain (mild, moderate & severe) is 66.9% (Tables-III), which is particularly high compared to the previous studies.^5^^,^^6^^,^^9^^,^^15^ It has even surpassed the prevalence of neck (58.2%) and shoulder pain (60.6%), ranked 6^th^ and 7^th^, respectively in terms of frequency. Mengestu and Zele^16^ found the same results, with a low back pain prevalence of 57.5%. They noted that lack of physical exercise, provisions of office at work and satisfaction with working environment were the factors associated with high prevalence of low back pain among teachers.^16^ Our study also showed a higher prevalence of low-back pain compared to the study conducted by Darwish and Zuhair among secondary school teachers in the Eastern region of Saudi Arabia (68.2% versus 63.8%).^6^ Our study augments the Darwish and Zuhair results that such a high prevalence of low back pain exists in this region of the world, considering we sampled almost the same study population. Furthermore, in our study, we were able to show that one third of our respondents (38.1%) who had low back pain, reported it as severe. In addition, we found out the significant association of the high prevalence of low back pain to Vitamin D deficiency, presence of chronic illness, feeling of anxiousness, and feeling of bad mood. Whether these factors are truly contributing the high prevalence of low back pain remains to be further verified, since low back pain was not found to be associated to the other variables such as magnitude of work, high number of classes taught, age, and BMI. 

After low back pain, knee pain was the next most frequent site of musculoskeletal pain found in our study, with a prevalence of 63.2%. Still, a 63.2% of prevalence is higher compared to previous similar study,^10^ more so 26.3% of the teachers reported that the pain is severe. And teachers at school for the handicapped, physical education teachers, kinder garden personnel, and school nurses were suffering from high prevalence of low back pain.^17^^,^^18^ Like among all categories of school teachers, 46% prevalence of low back pain was found in general population.^19^ Similar to the results on low back pain, we found significant associations of knee pain with the presence of a concomitant vitamin D deficiency, chronic illness, often feeling anxious and often feeling bad and their level of teaching at schools. 

Shoulder pain was documented in 59.2% of our study subjects. This is higher than the 48.7% reported by Yue in 2012,^9^ 55.9% in the Durmus study^10^ and 35.4% among nursery school teachers.^20^ On the other hand, neck pain was experienced by 56.8% of our respondents, better than the 69.3% reported by Chiu in 2007,^5^ but higher than the 42.1% reported by Darwish.^6^ Heel pain was documented in 56% of our respondents. Furthermore, upper back pain was reported by 47.5% in our study, which is fairly consistent with studies that showed at least a 50% prevalence of upper back and shoulder pain.^11^^,^^13^ Our prevalence of elbow pain (42%) is significantly higher than the prevalence reported by Darwish (10.0%).^6^ More so, our prevalence of wrist pain (40.5%) is also significantly higher than that reported by Darwish (16.2%).^6^ To our knowledge, our study is the first to report the prevalence of heel, hip joint and ankle pain among school teachers.

The factors that were found to be significantly associated with musculoskeletal pain among our respondents included a concomitant Vitamin D deficiency, presence of chronic illness, feeling of anxiousness, and feeling often bad mood (neck, shoulder, elbow, wrist, upper back, low back, hip joint, knee, ankle and heel pain), level of teaching (knee and ankle pain), marital status (hip joint and heel pain), body mass index (heel pain) (Table IV & V). Some of these findings (feeling of anxiousness & bad mood) were similar to those of other studies.^4^^,^^10^ We found no significant correlation of musculoskeletal pain with the number of teaching hours, teaching sessions, age and the duration of teaching, which were in contrast to the findings of the some of the studies reported in the literature. ^5^^.^^9^^,^^12^

As to the severity of musculoskeletal pain among our study subjects, low back pain topped our list with 38.1% of our respondent’s experienced severe pain, followed by knee pain (26.3%) and heel pain (24.1%). The high index of severity in these three anatomical areas is understandable since elementary and high school teachers spend most of their time standing while teaching. On the contrary, elbow pain, neck pain, wrist pain, ankle pain and knee pain had the highest percentage of mild pain (30.7%, 28.6%, 25.1%, 23% and 22.4% respectively). We cannot surmise our judgment on the prevalence of severity of pain considering this study was a survey, wherein the respondents subjectively assessed the pain on 10-point scale, they experienced in any of the anatomical parts, and not objectively measuring pain using any scale model such as the WILDA approach to pain assessment to standardize and optimize patient care.^21^

Overall, our study found very high prevalence of musculoskeletal pain among school teachers which is affecting their work, by missing out working days and eventually affecting the education system as a whole. An appropriate exercise program has to be adopted so as to alleviate pain and suffering among these school teachers. These programs could be incorporated into the system towards the health care needs of Saudi school teachers by the Ministry of Education of Saudi Arabia.

## Authors Contribution:

**AA **designed, organized and manuscript writing.

**AH** did literature review and responsible for all aspects of the study.

**AE & TH** involved in the development of study protocol, data collection & data entry.

**AJ **did statistical analysis and manuscript editing.

## Notes

***Limitations of the study: ***The limitations of this study were (i) subjective assessment of pain from the self- reported information provided in the questionnaire by the study subjects (ii) effect of confounding factors and (iii) subjects studied were only female school teachers.
